# The reliability and quality of YouTube videos as a source of breath holding spell

**DOI:** 10.1186/s13052-023-01570-0

**Published:** 2024-01-18

**Authors:** Mehmet Semih Demirtas, Nurettin Alici

**Affiliations:** 1 Faculty of Medicine, Department of Pediatrics, Aksaray University, Aksaray, Turkey; 2 Department of Social Pediatrics, Institute of Child Health, Hacettepe University, Ankara, Turkey; 3 Department of Pediatric Neurology, Ankara Training and Research Hospital, Ankara, Turkey

**Keywords:** YouTube, Breath holding spells, Cry, Infant, Children

## Abstract

**Background:**

Breath holding spells (BHS) are an important non-epileptic condition that is common in childhood and causes concern to families. YouTube is a powerful social media tool for accessing diseases and information such as BHS in child health. The aim of the study was to measure of the quality and reliability levels of the videos published in English on BHS uploaded on YouTube.

**Methods:**

The key words “infant”, “cry”, “breath holding spells”, holding spells” and “breath spells” were searched on the YouTube on November 14, 2022, in this study. Along with the general features of the videos, their quality and reliability were evaluated according to the global quality score (GQS), mDISCERN score.

**Results:**

Fifty-five videos were evaluated. The mDISCERN and GQS scores of the videos in the useful group were higher than those in the misleading group (*p* < 0.001, *p* < 0.001). In the useful group, 87.5% of academic institutions and 93.3% of medical doctors (MDs) uploaded high-score GQS videos, while this rate was 16.7% in independent users (*p* = 0.005). The positive correlation was found between mDISCERN and GQS scores (*p* < 0.001).

**Conclusion:**

The majority of YouTube videos on BHS contained useful information with sufficient quality. Professional associations such as universities and academic institutes need to produce better quality videos to provide families/users with more accurate and up-to-date information about BHS. We emphasize that YouTube should analyse videos published in the field of health, especially in the field of pediatrics, such as BHS, with committees consisting of expert health professionals, and publish them after evaluation. YouTube should consider collaborating with professional pediatrics health organizations such as American Academy of Pediatrics (AAP), academic institutes and universities in the field of BHS to produce high-quality videos.

## Background

Breath holding spells (BHS) is one of the involuntary non-epileptic conditions often accompanied by loss of consciousness, and its globally prevalence has been reported to be between 3–6% [1]. BHS usually occurs in response to a disturbing or surprising situation that affects the child physically or psychologically. Although the pathophysiology of BHS which presents with apnea and decreased Spo2 after breath-holding, postural tone changes, and myoclonic jerks, has not been fully elucidated, stimulus mechanisms such as autonomic dysfunction or vagal stimulation are prominent [2]. BHS seen in children aged 6 months to 4 years has three clinical forms: cyanotic, pale, and mixed type. Since BHS is usually a transient condition and is benign, there are not many articles in the literature on the subject [3].

Parents are very concerned about the BHS their children are experiencing, and it can lead to significant depression and stress, especially in mothers. The possibility of children having BHS again, as well as additional problems such as irritability and tantrums, prompts families to seek information and find solutions about BHS [4, 5].

As a result of the measures that started with the Covid -19 pandemic, lockdowns have occurred worldwide. In this context, internet and remote access programs have started to be widely used as mass communication tools in accessing information [6]. Families started to use the internet, especially the YouTube platform, as it became difficult to access physicians and health information through these sources due to the pandemic [7, 8].

YouTube is increasingly being used as a source of information on childhood diseases [8]. In our study, we aimed to examine the accuracy, quality and reliability of the videos, content and information about BHS on YouTube. As far as we know, this study is the first work in the literature that analyses the BHS videos of YouTube.

## Materials and methods

### Literature search

A detailed video search was conducted on YouTube (http://www.youtube.com) on 14 November 2022 using the keywords “infant”, “cry”, “breath holding spells”, holding spells” and “breath spells”. Since the majority of YouTube users prefer the “relevant” option in the video search [8, 9], 224 videos were found after the search with the relevant option. The videos in the study were examined and evaluated separately by 2 independent authors.

### Eligibility

Videos between 30 s and 20 min in English language were included in the study. Of the videos that were not included in the study, 94 of them were duplicate, 54 of them were irrelevant, 14 of them were non-English languages videos, 4 of them had audio and video problems and 2 of them were excluded because they did not comply with the specified time.

### Video features

The number of subscribers of the users who published the videos and the number of likes, dislikes, comments, views, daily views and duration times of the video in the study were included. The sources that uploaded the videos in the study were examined under 4 groups: academic institutions, medical doctors (MD), healthcare professionals other than MD and independent users. In addition, the target audience of the videos was categorized into 3 groups as parents, healthcare professionals and undefined.

### Evaluation of usability and quality/reliability of videos

Fifty-six videos identified within the scope of the Useful evaluation were analysed by the researchers in two parts as useful and misleading. Videos that contain correct and useful data about BHS and do not contain false and misleading data are useful; other videos have been described as misleading [3, 10].

The mMDISCERN reliability tool consisting of a total of 5 questions that can be answered as “yes” or “no”, which was used in previous similar health studies was used for the reliability and the GQS tool was also used for its flow and usability of the videos (Table 1) [8, 11].

**Table 1:** Modified DISCERN reliability and GQS quality tool

Modified DISCERN reliability tool	Global quality scale
1. Are the aims clear and achieved?	1. Poor quality, poor flow, most information missing, not helpful for patients
2. Are the sources of information reliable?	2. Generally poor, some information given but of limited use to patients
3. Is the information balanced and unbiased?	3. Moderate quality, some important information is adequately discussed
4. Are additional resources to information provided?	4. Good quality good flow, most relevant information is covered, useful for patients
5. Does the video address areas of controvesy/uncertainty?	5. Excellent quality and excellent flow, very useful for patients

### Ethics approval

Ethics committee approval was not obtained as in previous studies [7, 8, 10], because the study did not include human participation and the use of animal experiments, and information was obtained from a public platform.

### Statistical analysis

We used the SPSS v.22.0 program to analyse the data in the study. Shapiro–Wilk test was used to evaluate whether the data fit the normal distribution. Kappa test was used to evaluate the agreement between the two authors. Quantitative data that did not fit the normal distribution were expressed as median (min–max). The Mann–Whitney U test was performed for pairwise comparisons between “useful” and “misleading” videos. The relationship between two quantitative parametric variables was evaluated with spearman correlation. The Mann–Whitney U test was used to compare continuous variables between the two groups, as appropriate. The Kruskal Wallis test was used to evaluate quantitative variables between ≥ 3 groups. The Fisher’s exact was used for evaluating qualitative parameters. A *p* value less than 0.05 was considered statistically significant.

## Results

The study was conducted with a total of 56 videos, 40 (71.4%)1 of which were useful and 16 (28.6%) were in the misleading group. The median length of the videos was 4.06 min, 4.41 and 2.61 min in the useful and misleading group, respectively. The median values of daily views, comments and likes of the videos in the useful group were 5.22, 1.5 and 32.5, respectively; In the misleading group, this value was 6.4, 0 and 30.5, respectively. Other features of videos were summarized in Table 2.

**Table 2:** Evaluation of video features, mDISCERN and GQS scores

Variables	Useful *n* = 40	Misleading *n* = 16	*p*
**Video features, Median (Min–Max)**^**b**^			
Number of views	34006 (84–712453)	26201 (298–294140)	0.805
Number of likes	910.05 (0–20000)	128.62 (1–849)	0.353
Number of dislikes	0 (0–0)	0 (0–0)	0.828
Number of comments	33.85 (0–627)	18.38 (0–80)	0.588
Duration time (minute)	10.21 (0.46–27)	4.04 (0.45–14.56)	0.621
Number of per Daily views	196.45 (0.42–6459)	14.39 (0.59–89.2)	0.481
Number of subscribers	235380(150–2400000)	43630 (50–294000)	0.230
**Reliability and quality scores**^**b**^			
mDISCERN score (mean ± SD)	5 (4–5)	1 (1–2)	** < 0.001**
GQS (mean ± SD)	4 (4–5)	1 (1–2)	** < 0.001**
**Sources, n (%)**^**a**^			
Academic institutions/Universities	8 (20%)	0 (0%)	** < 0.001**
Physicians	15 (37.5%)	0 (0%)	
Healthcare professionals other than physicians	11 (27.5%)	3 (18.8%)	
Independent users (youtuber/parent)	6 (15%)	13 (81.3%)	
**Target audience, n (%)**^**a**^			
Health care professional	8 (20%)	0 (0%)	0.053
Patients/Parents	32 (80%)	16 (100%)	
**Video contents, n (%)**^**a**^			
Overwiev of BHS	17 (42.5%)	5 (31.3%)	** < 0.001**
Pathophysiology and general cautions	17 (42.5%)	0 (0%)	
Solution Techniques	4 (10%)	3 (18.8%)	
Examples	2 (5%)	8 (50%)	

^a^Fisher’s exact test was used

^b^Mann Whitney U test was used

When the video agreement between the two researchers was evaluated with the Kappa score, high agreement rates (0.921 and 0.928, respectively) was found for mDISCERN and GQS (*p* < 0.001, *p* < 0.001, respectively). mMDISCERN score, which is a video reliability score, was found to be 5 (4–5) in the useful group and 1 (1–2) in the misleading group (*p* < 0.001) (Table 2). When we evaluated videos in the misleading group, we have determined that the information in 4 videos is not reliable, the information in 5 videos is not correct, the source usage is not specified in 6 videos, and the information in 2 videos is biased.

Of the 40 uploaded videos in the useful group, 8 (20%) were uploaded by academic institutions, 15 (37.5%) by MDs, 11 (27.5%) from healthcare professionals other than MD, and 6 (15%) from independent sources. The target audience of 32 videos (%80) was parents and 8 (%20) of them were healthcare professionals. Considering the video content themes, 4 video (%10) had solution techniques, 2 video (%5) had examples and 17 videos explained overview (%42) and its pathophysiology (%42). In the misleading group, 13 (%81.3) of the videos were uploaded by independent users and 3 (%18.8) healthcare professionals other than MD (Table 2).

The mean GQS scores of the videos uploaded by academic institutions were remarkable higher than the other sources (healthcare professionals other than MDs, independent users (*p* < 0.001 and *p* = 0.001, respectively). The comparison of the reliability and quality scores according to video sources is summarised in Table 3.

**Table 3:** Comparison of the mDISCERN, GQS scores according to video sources

Reliability and quality scores	Academic institutions Universities *n* = 8 (14.3%)	Physicians *n* = 15 (26.8%)	Medical Sources^a^ *n* = 14 (25%)	Independent users *n* = 19 (33.9%)	p^*^
mDISCERN	5 (4–5)	4 (3–5)	3 (1–5)	2 (1–4)	** < 0.001**
GQS	5 (4–5)	4 (3–4)	3 (1–4)	2 (1–4)	** < 0.001**

^*^Kruskal Wallis test was used for evaluating, scores were given as median (min–max)

^a^Healthcare professionals other than physicians

When mDISCERN, GQS, daily views, likes, comments and subscriber numbers between the two groups were examined, a significant difference was found between mDISCERN and GQS scores (*p* < 0.001, *p* < 0.001, respectively), but no significant difference was found between other variables.

When the GQS scores were reclassified (4–5 score high quality; 3 score medium quality; 1–2 score low quality) in the useful group, it was found that 32 videos (80%) were of good quality, 6 (15%) were of medium quality, and 2 (5%) were of low quality. In addition, 7 out of 8 (87.5%) videos uploaded by academic sources and 14 out of 15 (93.3%) videos uploaded by MDs were found to be of high quality, while 4 out of 6 (66.7%) videos uploaded by independent institutions were found to be of medium quality and 1 of them (16.7%) was of high quality (*p* = 0.005).

When the factors affecting the number of daily views (like, comment, number of subscribers, duration, mDISCERN, GQS) were evaluated by correlation analysis, it was determined that the number of subscribers, comments and duration time were effective (*p* < 0.001, *p* < 0.001, *p* = 0.006, respectively) (Table 4). The positive correlation was found between mDISCERN and GQS scores (*p* < 0.001).

**Table 4:** Correlation of video features, GQS and mMDISCERN scores

	Number of per daily views	Number of likes	Number of comments	Duration Time	mDISCERN score	GQS score	Subscriber numbers
Number of per daily views							
Pearson Correlation	1	0.298^a^	0.855^b^	0.414^b^	0.035	-0.030	0.612^b^
Sig. (2 tailed)		0.26	** < 0.001**	**0.002**	0.799	0.828	** < 0.001**
N	56	56	56	56	56	56	56
Number of likes							
Pearson Correlation	0.298^a^	1	0.230	0.460^b^	-0.053	-0.047	0.679^b^
Sig. (2 tailed)	0.26		0.089	** < 0.001**	0.696	0.728	** < 0.001**
N	56	56	56	56	56	56	56
Number of comments							
Pearson Correlation	0.855^b^	0.230	1	0.477^b^	-0.001	-0.039	0.523^b^
Sig. (2 tailed)	** < 0.001**	0.089		** < 0.001**	0.995	0.773	** < 0.001**
N	56	56	56	56	56	56	56
Duration Time							
Pearson Correlation	0.414^b^	0.460^b^	0.477^b^	1	0.184	0.144	0.384^b^
Sig. (2 tailed)	**0.002**	** < 0.001**	** < 0.001**		0.174	0.290	**0.003**
N	56	56	56	56	56	56	56
mDISCERN score							
Pearson Correlation	0.035	-0.053	-0.001	0.184	1	0.928^b^	-0.084
Sig. (2 tailed)	0.799	0.696	0.995	0.174		< 0.001	0.540
N	56	56	56	56	56	56	56
GQS score							
Pearson Correlation	-0.030	-0.047	-0.039	0.144	0.965^b^	1	0.049
Sig. (2 tailed)	0.828	0.728	0.773	0.290	** < 0.001**		0.724
N	56	56	56	56	56	56	56
Subscriber numbers							
Pearson Correlation	0.612^b^	0.679^b^	0.523^b^	0.384^b^	0.056	0.049	1
Sig. (2 tailed)	** < 0.001**	** < 0.001**	** < 0.001**	**0.003**	0.682	0.724	
N	56	56	56	56	56	56	56

^a^Correlation is significant < 0.05 level

^b^correlation is significant at the 0.01 level (2-tailed)

^c^Spearman correlation was used for evaluating

## Discussion

This article is the first study in the literature examining the videos made on YouTube on BHS. We determined that mMDISCERN and GQS score were significantly higher in useful group than misleading group (*p* < 0.001). In the Useful group, 87.5% of academic institutions and 93.3% of MDs uploaded videos with a high GQS (4–5 score), while this rate was 16.7% from independent sources (*p* = 0.005). In the correlation analysis between the number of daily views, number of like, comment, subscribers, duration time, mDISCERN, GQS score parameters, we found that number of subscribers, comments and duration time were effective on the number of daily views (*p* < 0.001, *p* < 0.001, *p* = 0.006, respectively).

It has become a necessity for social media/internet users in all segments of society to access the accurate and reliable information they want to learn in this age of rapidly increasing access to information [12, 13]. In addition to the ubiquity and easy access of social media, the use of the internet/social media is increasing due to the impact of the COVID-19 pandemic lockdown [14]. Social media has the advantage of providing advice and guidance by connecting users with similar health problems or demands around the world. YouTube, which is the second most preferred among the social media platforms, is an easily accessible and central content source, is highly preferred in individual searches in the field of health due to its video content and narration [15, 16]. Information obtained from sources on social media opens doors for users to access accurate or inaccurate health information [17]. Similar to the rate of videos in the useful category in studies [18–20] on YouTube and health in the literature, 71.4% of the videos in the study were evaluated in the useful category. The use of YouTube as a source of information by health professionals increases the quality and reliability of the videos in the videos related to BHS.

There is no supervisory control mechanism over which users can upload health videos to YouTube and the advice or medical claims contained in the videos. This situation causes the accuracy of the information to become accessible without being analysed and approved. It is important for parents to be aware of the quality and accuracy of the medical information about children they access online [21, 22]. In a study in which was evaluated YouTube videos about hip arthritis, it was shown that the diagnostic or treatment information contained in about 85% of the videos was of poor quality [23]. In another study evaluating PSA related YouTube videos, it was shown that 73% of the videos were of poor quality [24]. 80% of the videos evaluated in the Useful category consisted of videos that were useful to users with information content with high GQS scores. This high rate is due to the increase in the number of health videos uploaded by academic institutions, MDs and healthcare professionals other than MD [25]. In addition, the majority of the videos uploaded by academic institutions and MDs (87.5% and 93.3%, respectively) were of high quality compared to other groups (*p* = 0.005). This result shows that videos broadcasted by health professionals with theoretical and practical knowledge in the field of health have better information content and flow. This result also shows that social media users/parents who want to access accurate information on BHS can do so through videos published by MDs and academic institutions.

BHS can cause severe anxiety in parents or extreme fear of the child's sudden death or future development of a mental abnormality. As a result of this concern, families want to reach the most accurate and solution-oriented information for their children as soon as possible [26, 27]. Easily accessible social media platforms such as YouTube are among the first sources families use to get information [28]. Videos on YouTube include the number of views, comments, likes and dislikes, and in a general video search, the searched words are sorted according to their relevance. Instead of watching videos based on content and quality, users usually watch videos that appear in the first 3–4 pages of search [7, 8]. In similar pediatric studies in the literature, it has been shown that the quality and flow scores of videos in the useful category are higher [29, 30]. In this study, the mDISCERN and GQS scores of the videos evaluated in the useful category were found to be significantly higher, similar to the literature (*p* < 0.001, *p* < 0.001). While the video quality and content are generally evaluated in YouTube videos made in the field of health, the factors that cause the videos to be watched more have not been analysed much [7, 31]. The most remarkable finding we found in our study was that as a result of the analysis of the factors affecting the number of daily views of BHS videos, the number of subscribers, comments and duration time were effective on daily views (*p* < 0.001, *p* < 0.001, *p* = 0.006, respectively). This result shows that videos broadcasted by channels with organised video titles, open to comments, short video duration, and high number of members attract more attention of parents/users and increase video viewing rates. In addition, when the most watched videos were re-evaluated, the fact that the majority of these videos were broadcast by channels providing information about mother–child-baby was another issue that caught our attention.

## Conclusion

This current study demonstrated that the vast majority of BHS videos (71.4%) uploaded to YouTube, along with technological and social media developments, present useful information for parents/users. Moreover, 80% of the videos were of good quality, and the quality of videos uploaded by academic institutes and MDs was higher (87.5% and 93.3%, respectively). In this regard, we recommend that organizations such as the World Health Organization (WHO), the American Academy of Pediatrics (AAP), universities, medical doctors and academic institutes share more videos on YouTube to provide accurate and up-to-date information about BHS and treatment options. Professional pediatric organizations such as the AAP and National European Paediatric Societies and Associations (EPA/UNEPSA) should also consider collaborating with YouTube to create high-quality health content. We emphasize that YouTube's videos on health-related issues, especially child health and diseases such as BHS, should be evaluated by committees of expert health professionals and published after evaluation. We think that showing the most watched and popular videos at the top of YouTube is a handicap in accessing accurate information in the field of health. Therefore, YouTube should ensure that videos produced by reliable sources such as universities, academic institutes and AAP are ranked first in video searches made by parents/users. In addition, YouTube may plan to ban/unpublish videos with bias and misinformation using reliable verification criteria such as mDISCERN and GQS on BHS and health videos.

## Data Availability

Not applicable.

## Abbreviations

MD: Medical Doctor
BHS: Breath Holding Spells
GQS: Global Quality Scale
mDISCERN: Modified DISCERN score
WHO: World Health Organisation
PSA: Prostate specific antigen
AAP: American Academy of Pediatrics
EPA/UNEPSA: National European Paediatric Societies and Associations
